# María Jesús Santesmases. *The Circulation of Penicillin in Spain: Health, Wealth and Authority*

**DOI:** 10.1093/jhmas/jrab047

**Published:** 2021-12-27

**Authors:** Cristina Moreno Lozano

**Affiliations:** University of Edinburgh, United Kingdom

**Keywords:** Penicillin, Biomedicine, Pharmaceuticals, Gender, Circulation, Dictatorial Spain

In her book *The Circulation of Penicillin in Spain: Health, Wealth and Authority*, María Jesús Santesmases describes the entry of penicillin into Spain, and its installation within Spanish medical and public cultures during the early years of the Francoist dictatorship. The book considers the development of biomedical knowledge, scientific and clinical practices, and pharmaceutical manufacturing procedures around penicillin. The book not only follows penicillin’s history in a local context; by tracing its circulation, Santesmases also pays attention to the complex geopolitical and cultural circumstances that informed the Spanish adoption of antibiotic therapies.

Penicillin had to navigate a tumultuous socio-political context in post-Civil War Spain. At the start, Santesmases describes how penicillin entered the country through international trade negotiations among the dictatorship, international authorities, and markets, primarily in the United Kingdom and the United States, as well as through smuggling (Chapters One and Four). Government authorities formed an expert committee that would assess and control the medical administration of the drug received through legal imports, while “blisters” of penicillin were kept illegally in the refrigerators of pubs and restaurants in Madrid, ready for purchase to those with enough money and social networks. Scarcity, rationing, and regulation are key features of this history.

Both its scarcity and the sensational accounts of its healing attributes, Santesmases suggests, contributed to the growing cultural perception of penicillin as a miracle drug, and an icon of scientific progress and hope for a plausible better (and healthier) future in dictatorial Spain. In his 19-day visit to Spain in 1958, Alexander Fleming (Chapter Two), the British scientist who identified the microorganism that produced penicillin, was welcomed as a hero by both the Spanish people and press. As if he were bringing penicillin in his own hands, Fleming was met with devotion by the masses in the streets of Barcelona, Madrid, and Seville—becoming a key asset for Franco’s dictatorial propaganda. Santesmases suggests that Fleming’s visit could be seen as an example of the growing symbolic power of antibiotics that was beginning to circulate in Spain at the time, even when the majority of citizens had not yet experienced the therapeutic effects of this drug.

In this monograph, antibiotics serve as a lens through which the reader can get a glimpse into the history of dictatorial Spain. Historians of science have recently offered counter-arguments to mainstream historiographies of Spain’s economic development that emphasise the “tardiness” of its industrialisation due to the authoritarian government. Santesmases offers an analysis of the regime not as a monolithic authoritative actor, but as a fragile alliance of communities with differing (political and economic) interests, where scientific and technological projects played important roles in giving shape to health, wealth, and authority in the country, as the title of the book suggests.

The book will be particularly relevant for historians of medicine and biomedical sciences interested in the gendered and geopolitical distribution of science. Throughout the book, the author weaves a complex narrative, which includes often-unacknowledged actors, including many women. This comes across particularly in her description of the factory-laboratory model in the early antibiotic manufacturing plants (Chapters Three and Five). A state-controlled market was officially stablished at the start of the 1950s, when the two pharmaceutical companies, *CEPA* and *Antibióticos S.A*., launched shortly after penicillin made its entry into Spain. Although these companies began as bottling plants for imported penicillin, soon they would implement full drug manufacturing and incorporate a research division.

Through a “collective biography” approach, Santesmases argues that it was mostly young women who were employed as factory-line workers in the incipient antibiotic pharmaceutical industry of the time, whilst men performed senior roles in management, research and technical support (with a few exceptions, such as lab technician Sagrario Mochales). To me, the factory-line women workers in Santesmases’s book resemble the story told in Mar Hicks’s (2018) *Programmed Inequality: How Britain Discarded Women Technologists and Lost Its Edge in Computing.* As for women technologists, the factory-line tasks performed in bottling penicillin blisters were conceived as being primarily manual and artisanal, and largely “unskilled.” Screening required what then could have been conceived as feminine traits of careful and patient engagement with materials. Santesmases’s use of visual historical resources, as well as oral history, helps to not just expose the “invisible” and gendered work in the history of penicillin in Spain, which is not always available in the standard archives and textual accounts, but also to weave a complex narrative. In fact, I would have liked to hear more on the methods and process of reconstructing such a collective gendered history, which are regrettably not thoroughly discussed.

Throughout the book, Santesmases uses the concept of “circulation” as an analytical tool, inspired by the concept of “trafficking,” proposed by Maria Rentetzi (2008).^1^ Both Rentetzi and Santesmases emphasise that the focus of their research is in the actual *movement*, be it the *trafficking* or the *circulation*, of scientific materials, their identities in transition, and the cultures that support these movements.

In this book, the notion of circulation is employed to investigate how penicillin underwent “journeys from promise to materiality, from dreams to cures, (…) constructing its identity as a transnational object in the healing cultures of the mid- to late twentieth century” (p. 197). Circulation is thus used to complicate simple narratives of inevitable progress and dissemination, and account for how circulation itself contributed to making of penicillin a powerful and symbolic medicine.

The book closes by describing the trajectory of the emergence of antimicrobial resistance as an issue of concern in medical scientific papers and public policies until the early 1980s (Chapters Six and Seven). The author demonstrates that knowledge of antimicrobial resistance—and resistance itself—indeed circulated widely in late-twentieth century Spain as much as overseas, laying the grounds for screening projects, regulatory reforms, policies for surveillance of antibiotic use, and laboratory experiments to find newer antibiotics. Santesmases invites us to continue this history of antibiotics through further analysis of the local forms of the circulation of antimi crobial resistance and knowledge about resistance.

## Footnotes

1 Maria Rentetzi, *Trafficking Materials and Gendered Experimental Practices: Radium Research in Early 20^th^ Century Vienna* (New York: Columbia University Press, 2008). On the notion of “circulation,” see also María Jesús Santesmases and Christoff Gradmann, “Circulation of Antibiotics: An Introduction,” *Dynamis* 31 (2011): 293–303.

